# On the Application of Gutta Percha in the Treatment of Diseases of the Skin

**Published:** 1853-07

**Authors:** Robert I. Graves


*On the Application of Gutta Percha in the Treatment of Diseases of the Skin.* By Robert I. Graves, M. D., F. R. S.—Dr. Graves directs the attention of practitioners, in the treatment of certain forms of cutaneous disorder, to the necessity of protecting the abraded and tender surface of the skin, the *cutis vera*, from the contact and action of the air and other hurtful agents. The effect of blood coagulating over a cut or abraded surface is known to be beneficial, and not less serviceable are those secretions which are furnished by surfaces abraded and in a state of inflammation. The premature and forcible removal of all these discharges, which are to be regarded as natural coverings, is always injurious, and tends to continue inflammation and irritation, and consequently to defer healing and protract the disease.

Dr. Graves thinks that artificial substitutes for these secreted coverings, or artificial coverings over them, may be useful in effecting the healing of extensively abraded surfaces in cutaneous disorders. Of this nature are solutions of collodion, which have been in occasional use for some time; and more recently solutions of gutta percha in chloroform have been employed with similar beneficial results.

It is well known that there are certain obstinate forms of cutaneous disorder, in which, whether from the nature of the disorder, or the extent of skin abraded, denuded, and irritated, it is often difficult to effect either cure or alleviation. Such are some forms of psoriasis, eczema, impetigo, and leprosy. In treating affections of this kind, a covering of gutta percha seemed to Dr. Graves well calculated to protect the tender surface, and thereby to promote the healing process. After various trials with this agent, the results have been such, that Dr. Graves conceives that they ought to be well known. The therapeutic application of gutta percha is mentioned by Dr. Neligan in his recent work. But it may be useful here to give some notice of the method from the account by Dr. Graves himself.

When the saturated solution of gutta percha in chloroform is spread by means of a camel's hair pencil over a portion of the skin, the solvent fluid rapidly evaporates, leaving a delicate and extremely thin pellicle of gutta percha firmly adhering to the part. The peculiar toughness of gutta percha prevents this pellicle from being brittle, and therefore it is much less liable than collodion to crack and fall off in small scales. On the forehead or face, where it is not affected by friction of the clothes, it remains firmly attached for five or six days, or even longer; but on other portions of the surface it is often rubbed off much sooner. Over dry eruptions of the skin it lasts longer than over those which are moist, and over smooth and firm spots, of course, longer than over those covered with rough morbid scales or loosely adhering crusts. Before the application of this solution, therefore, the practitioner will do well to render the portions of the cutaneous disease to which he intends to apply it, as free as psssible from crusts or scales, by means of poultices, alkaline lotions, &c. When the precaution is taken, he will find that the artificial cuticle which he has applied with his brush will in certain cases act most sensibly on the subjacent disease, diminishing inflammation and its consequences, and powerfully contributing to the restoration of the healthy structure of the skin.

Whether it acts by the equable pressure which the film of gutta percha, suddenly coagulated from the fluid state, must exert on the surface to which it becomes so firmly adherent, or whether its efficacy be rather owing to other causes, it is beside the object of Dr. Graves in writing this notice to discuss. He remarks, however, that the curative properties of such an artificial covering are not confined to the skin; for, in the "Gazette Medicale de Paris" (April 10, 1852), is an account of the successful application, by M. Dechange, of collodion in orchitis; and the *modus operandi* is said by him to consist in its compressing the tissues, and protecting the parts from the action of the air, which he thinks is a powerful element of phlegmasia. Without giving an unqualified assent to this explanation, Dr. Graves has no hesitation in announcing his firm conviction, that, in diseases of the skin, this solution furnishes us with a new and active remedy, exerting effects quite different from those produced by any topical application hitherto in use.

The transparency of this artificial membrane enables the surgeon to watch the progress of the subjacent diseased skin, and its colorless nature prevents it from disfiguring the face when the eruption occupies that part. Its perfect cleanliness, too, is no small advantage, and affords a very agreeable contrast when compared with the usual ointments, &c.

The observations of Dr. Graves confirm what reasoning on this subject would lead us to expect, that this application is more suited for dry, scaly, tubercular, and chronic diseases of the skin than for acute affections attended with much oozing of fluid and comparatively active inflammation.

Still, its good effects are by no means limited to chronic diseases of the skin, or those of a scaly, dry nature; for, as will hereafter appear, Dr. Graves has seen it decidedly useful in the spreading form of impetigo. His experience of this remedy makes him anxious to witness its application in the first stages of erysipelas, as analogy leads him to hope for good results in such cases.

Of course the patient must aid the efforts of the physician, and must, as far as possible, abstain from every thing which tends to rub off or injure the artificial cuticle; for its virtue ceases when its continuity is broken and the external air finds admission to any part of the diseased surface.

Early in the month of November, 1851, Dr. Graves was called to visit Mrs. C., from ———. She was about fifty years old, full and plethoric; the mother of a large family, and, until the disease of which she then complained, commenced, generally healthy. About two years before, she observed small spots of impetigo on her limbs and body, which succeeded each other, some healing while fresh ones appeared. During summer she was nearly free from them, but last autumn they returned with greater virulence than ever, and have since increased both in size and numbers, some being larger than the hand, and attended with constant oozing of fluid, which imperfectly coagulates, forming loose and thin crusts. The itching at night was intolerable, and nearly deprived her altogether of sleep. Dr. Graves employed the usual general and topical treatment for a fortnight without alleviation of her sufferings, when he thought of trying the saturated solution of gutta percha in chloroform, and had it carefully applied by Mr. Nicholls of Dawson Street, at first by way of trial, to one of the smaller spots, and on the following days to each of the larger patches of eruption in succession. The relief obtained was such that it appeared almost incredible, both to the patient and her family. Her cure was accomplished in less than three weeks; for, dreading the sudden stopping of so great a discharge and so much cutaneous irritation, he proceeded cautiously, and towards the end of the cure, when she returned home, he directed an issue to be inserted in her arm, as a measure of precaution. She has continued well up to the present time (11th April, 1852).

In this patient the attendants were at first obliged to re-apply the gutta percha every second day, as it was rapidly detached and broken up into large flakes by the discharge from the subjacent surface. Its healing influence, however, speedily diminished the diseased secretion, and then, the artificial cuticle remained longer adherent, and it was not necessary so often to use the scissors for the purpose of cutting off the loose portions of gutta percha membrane previously to applying a fresh layer. The camel's hair brush should be plunged, the moment it has been used, into hot water, to prevent it from being consolidated by the coagulated gutta percha.

This case caused a great sensation among the patient's friends and relatives, and many were the inquiries made relative to the method of cure employed. Dr. Graves confesses that his own astonishment at the result was not less than theirs.

Since that time Dr. Graves has repeatedly used this application in *acne of the face*, in which disease each of the pimples should be covered with the solution, and the patient enjoined not to rub off the pellicle by washing, &c.

In some this treatment alone causes a material and rapid diminution of this tormenting eruption, and by perseverance in this plan, there is every appearance in two of his patients that the tendency to throw out the pimples is gradually ceasing.

Finally, in several cases of psoriasis Dr. Graves applied this solution with great benefit. In this disease much care must be taken to prevent the application being rubbed off by the clothes, and no woolen stockings or rough garment of any sort should be allowed next the skin. I had the satisfaction of curing in a fortnight a chronic psoriasis of the back of the hands and arms in a lady who had been under homœopathic treatment for six months without deriving the least advantage from the infinitesimal doses prescribed by the practitioner.

Since the preceding observations were written, Dr. Graves received the following note from Mr. Moore. The additional information concerning the effects produced by the application of collodion strongly verifies the observations respecting the local antiphlogistic action of a gutta percha pellicle :

" M. La Tour has for some time advocated the doctrine that external inflammation may be speedily subdued by withdrawing the affected surface from the influence of the air. This he effects simply by a layer of collodion, which, he says, rapidly assuages the inflammation of gout and articular rheumatism. At the meeting of the Academy of Medicine, on the 8th of February, M. La Tour related a case of peritonitis in which the symptoms were dissipated within twenty-four hours, by the application of a layer of collodion to the whole surface of the abdomen."

POSTSCRIPT, *June* 7, 1852.

My anticipation respecting the utility of an artificial cuticle applied over the parts affected by commencing erysipelas, has, I find, been verified, as appears from the following paragraph taken from Dr. Neligan's able Treatise on Diseases of the skin:

"Acting as an impermeable varnish, and probably producing some effect, also, by the compression it causes, collodion has been successfully employed by Spengler and Rapp, as a local application in erysipelas. The parts are thickly coated with it by means of a camel's hair pencil and it is renewed as often as may be required in consequence of its cracking and peeling off when dry."

When Dr. Stokes heard of the success of Dr. Graves in other cases, he resolved to try the gutta percha solution in small-pox, and the result of two trials is most encouraging, and leads to the hope that at length a means of preventing the formation of disfiguring scars on the face in that disease has been discovered. Dr. Stokes has allowed Dr. Graves to publish the two following cases treated by him at the Meath Hospital, and noted by this clinical clerk, Mr. Robert P. White.

Anne Kenny, aged eighteen, was admitted into the Meath Hospital, 11th May, 1852. She was never vaccinated; her illness began on the 6th of May by rigors, headache, and pain in her loins, but without any vomiting. On the 8th, the eruption appeared, and on coming into hospital, the eruption was well formed and confluent on her face; the fever of a typhoid character, with considerable prostration of strength; she was much annoyed with pain and itching of her face. She was ordered wine and carbonate of ammonia mixture, and her face to be painted with a solution of gutta percha in chloroform.

The solution was applied with a soft brush; the entire face being well coated with it, and after an interval of a few minutes (just sufficient to allow the previous coating to dry), a second coat of the solution was applied.

This application, she said, gave her great relief, and allayed the pain and itching. On the 13th she was much better, and the solution was again applied in the same manner.

22d May. She has proceeded most favorably; all the crusts have come off the face in large pieces, and there is hardly any trace of the disease remaining except a slight discoloration. During the whole time of her illness, from the application of the solution, her face continued moist, and there was no ulceration in any place.

Catherine Sherlock, aged twelve, was admitted 13th May (seventh day of eruption). Her illness commenced by headache, pain in her loins, and rigors, but without sickness of the stomach; she was never vaccinated. On admission she had a good deal of fever of a typhoid type; the eruption had come out well, was confluent, and had completely covered her face; she complained very much of pain and itching of her face and head, which kept her from sleeping. The solution of gutta percha was applied as in the former case, and apparently gave her much relief, for she was easier afterwards, and ceased rubbing and tearing her face in the manner in which she had been doing before. Wine was also freely used as in Kenny's case.

22d May. All trace of the disease has nearly gone, the crusts coming off in large pieces, and the remaining marks of the disease being very trifling when compared with the violence of the attack.

The same effect of the solution in keeping the face moist was also observed in this case as in the preceding.

In communicating these cases, Dr. Stokes observed, as worthy of notice, and probably connected with the beneficial result produced, that the most remarkable effect of the gutta percha was to keep the face constantly moist, and to prevent the formation of hard and irritating crusts. He also mentioned a singular illustration of the effects of total exclusion of air from the cutaneous surface as a preventive of the eruption in small-pox. It was that of a man who, while in the Meath Hospital for a scrofulous enlargement of the knee-joint, was attacked with this disease; the knee had been previously tightly strapped with adhesive plaster, and on the disappearance of the eruption, it was seen, on removing the strapping, that not a single pustule had been developed on the parts which were thus covered.

It is scarcely necessary to add, that however useful solution of gutta percha in chloroform, or any other topical applications, may prove, it would be, nevertheless, absurd to expect from them a radical cure of those chronic cutaneous affections which arise from constitutional causes, whether strumous, syphilitic, or cancroid. As well might we look for the cure of scarlet fever, smallpox, or measles, by means of remedies applied to the skin solely with the view of relieving the eruption. Still, in every chronic disease, it is manifestly desirable to diminish, by topical means, the annoyance or disfigurement arising from the cutaneous affection. This relief cannot but be serviceable if it did nothing else than afford time for the due and gradual employment of a well-directed constitutional treatment. Dr. Graves saw this lately well exemplified in the case of a young lady who had been under the care of an eminent London surgeon for many months, and from whose medicines she had derived no relief. She was the subject of a most disfiguring eruption, agreeing in character with the *lupus supiginosus* of late writers; it occupied sparsely the nostrils and cheeks, but on the forehead it formed an extensive group of sores. Some spots it left hypertrophied after healing, but in general its course was marked by white depressed seams. It had been the seat of constant irritation, which the patient could not avoid increasing by scratching during sleep, and consequently the surface of the face usually exhibited a number of bloody points, adding to its disagreeable appearance. The entire eruption healed after a six weeks' application of the gutta percha, and she is now (what she had not been for two years) free from any cutaneous irritation. Time alone can determine whether relief will be permanent; but there can be no doubt of the great value of the advantage already gained.

Dr. Graves observes that, in psoriasis and other chronic cutaneous complaints unattended with any constitutional derangement, it is of the greatest consequence to check the growth of each new spot. This the gutta percha does most effectually in psoriasis, and when applied daily to any recent points of irritation, it smothers, as it were, each nascent centre of future blotches.—*Dub. Quart. Journ.*

